# Extracts from Traditional Chinese Medicinal Plants Inhibit Acetylcholinesterase, a Known Alzheimer’s Disease Target

**DOI:** 10.3390/molecules21091161

**Published:** 2016-08-31

**Authors:** Dorothea Kaufmann, Anudeep Kaur Dogra, Ahmad Tahrani, Florian Herrmann, Michael Wink

**Affiliations:** 1 Institute of Pharmacy and Molecular Biotechnology, Department of Biology, Ruprecht Karls University Heidelberg, Heidelberg 69120, Germany; mad.rani@gmail.com (A.T.); Florian.Herrmann@t-online.de (F.H.); wink@uni-hd.de (M.W.); 2 Centre for Pharmacognosy and Phytotherapy, The School of Pharmacy, University of London, 29-39 Brunswick Square, London WC1N 1AX, UK; anudeep.k.dogra@gmail.com

**Keywords:** acetylcholinesterase, acetylcholinesterase inhibitor, isoquinoline alkaloids, berberine, coptisine, palmatine, Alzheimer’s, *Coptis*, natural products, Traditional Chinese Medicine

## Abstract

Inhibition of acetylcholinesterase (AChE) is a common treatment for early stages of the most general form of dementia, Alzheimer’s Disease (AD). In this study, methanol, dichloromethane and aqueous crude extracts from 80 Traditional Chinese Medical (TCM) plants were tested for their in vitro anti-acetylcholinesterase activity based on Ellman’s colorimetric assay. All three extracts of *Berberis bealei* (formerly *Mahonia bealei*), *Coptis chinensis* and *Phellodendron chinense*, which contain numerous isoquinoline alkaloids, substantially inhibited AChE. The methanol and aqueous extracts of *Coptis chinensis* showed IC_50_ values of 0.031 µg/mL and 2.5 µg/mL, therefore having an up to 100-fold stronger AChE inhibitory activity than the already known AChE inhibitor galantamine (IC_50_ = 4.33 µg/mL). Combinations of individual alkaloids berberine, coptisine and palmatine resulted in a synergistic enhancement of ACh inhibition. Therefore, the mode of AChE inhibition of crude extracts of *Coptis chinensis*, *Berberis bealei* and *Phellodendron chinense* is probably due to of this synergism of isoquinoline alkaloids. All extracts were also tested for their cytotoxicity in COS7 cells and none of the most active extracts was cytotoxic at the concentrations which inhibit AChE. Based on these results it can be stated that some TCM plants inhibit AChE via synergistic interaction of their secondary metabolites. The possibility to isolate pure lead compounds from the crude extracts or to administer these as nutraceuticals or as cheap alternative to drugs in third world countries make TCM plants a versatile source of natural inhibitors of AChE.

## 1. Introduction

With more than 46 million people suffering from Alzheimer’s disease (AD) worldwide [1], this neuro-degenerative disorder is the most common form of dementia in elderly people [2]. The progressive degenerative brain syndromes connected to dementia affect memory, thinking, behaviour and emotion. Typical symptoms include loss of memory and difficulties to perform previously routine tasks. Patients also have problems with finding the right words or understand what people are saying and they often undergo personality and mood changes [3]. During the progression of AD, the death of nerve cells in the cerebral cortex leads to a shrinkage of the brain. In consequence, gaps develop in the temporal lobe and hippocampus, where new information is stored and retrieved. These lesions affect the ability to remember, think, speak and make decisions [3].

Brain processing speed and memory is determined by the neurotransmitter acetylcholine (ACh). A low level of ACh results in impaired learning and memory as well as general “slow thinking”. Furthermore, a deficiency of ACh seems to be directly correlated to AD [4]. The level of ACh in the brain of patients suffering from AD is greatly reduced compared to the healthy patients. In late stages of AD, levels of ACh have declined by up to 85% [5]. This decrease results from the inability to synthesise enough ACh for sufficient transmission of information. Furthermore, ACh is broken down in the synaptic cleft by acetylcholinesterase (AChE). Therefore, one way to elevate ACh levels in the brain is to inhibit AChE. The breakdown of ACh is decreased and more ACh is available to bind to ACh receptors resulting in an improvement of cognitive function [6,7]. AChE inhibitors are still the first choice of drugs for the treatment of AD. The AChE inhibitors galantamine and rivastigmine are used in mild to moderate stages of the disease; donepezil is the drug of choice for all stages. This therapy is no longer considered to be only symptomatic, but also disease modifying [8,9]. Another option is memantine, which is an antagonist of the *N*-methyl-d-aspartate receptor and prescribed for moderate to severe cases of AD either as single compound or in combination with donepezil. Although all of these therapies improve the length and quality of life of the patients, they are only symptomatic and fail to cure the disease [10,11].

Various plant-derived compounds are already used for the treatment of AD. The most prominent examples are physostigmine, galantamine (Reminyl^®^) and huperzine A, but more than 150 different plant species in various preparations and mixtures have been used in the context of age related CNS disorders [12,13,14,15]. We have also already showed the anti-AChE activity of myrtenal, a monoterpene derived from *Taxus baccata* [16]. Therefore, it can be assumed that plants are still a promising source of new bioactive compounds with anti-AChE activity.

This study investigates the use of plants from Traditional Chinese Medicine (TCM), a complete medical system used to diagnose, treat and prevent illness for thousands of years, as inhibitors of AChE. Eighty of the most commonly used TCM plants were tested for their in vitro inhibitory activity of AChE. Contrary to the approach of isolating single compounds from plants our idea was to use complex extracts. These consist of a wide variety of different secondary metabolites, usually belonging to different chemical classes. These chemical compounds can interfere with their targets in a pleiotropic manner. The overall effect is sometimes not only additive, but even synergistic. This means that the overall effect of a mixture is greater than the sum of the individual effects [17,18].

We were able to show that three of the TCM plants, which contain isoquinoline alkaloids, substantially inhibited AChE. The most remarkable finding was that the alkaloid containing methanol extract of *Coptis chinensis* showed a 100-fold more powerful AChE inhibition than galantamine. The mode of action of the highly active extracts is probably due to synergistic interactions, which could be shown when individual alkaloids, such as berberine, coptisine and palmatine (which occur in the extracts) were combined.

## 2. Results

### 2.1. Inhibition of Acetylcholinesterase by Extracts from TCM Plants

In this study methanol, dichloromethane and aqueous crude extracts from 80 TCM plants were tested for their in vitro anti-acetylcholinesterase activity. Physostigmine and galantamine, both known acetylcholinesterase inhibitors [19], were used as the positive controls. The extracts of *Berberis bealei* Carrière, Berberidaceae (formerly *Mahonia bealei*; Shi Da Gong Lao), *Coptis chinensis* Franch, Ranunculaceae (Huang Lian) and *Phellodendron chinense* Scheid., Rutaceae (Huang Bai) showed the highest inhibition of AChE activity. None of these extracts was cytotoxic in COS7 cells at their respective AChE inhibitory concentrations (Table 1) suggesting their potential therapeutic application. A high ratio between the IC_50_ in COS7 cells and corresponding AChE inhibition denotes a beneficial therapeutic profile of the compound. IC_50_ values for all other plant extracts are listed in Table 2.

### 2.2. Phytochemical Analysis of Most Active Extracts

**Figure 1:**
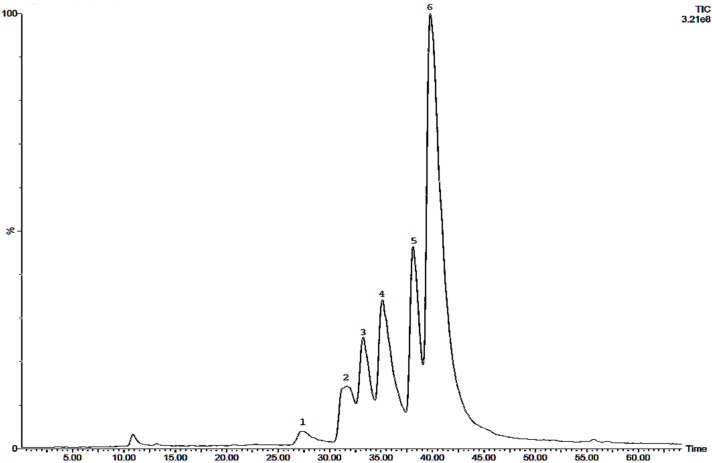
HPLC-MS total ion current chromatogram of a methanol extract of *Coptis chinensis*. Peaks: 1: Tetradehydroscoulerine / tetrahydrocheilanthifolinium (*m*/*z* = 322); 2: columbamine (*m*/*z* = 338); 3: epiberberine (*m*/*z* = 336); 4: coptisine (*m*/*z* = 320); 5: palmatine (*m*/*z* = 352); 6: berberine (*m*/*z* = 336).

Literature lists the alkaloids berberine, coptisine and palmatine as the main compounds of *Berberis bealei* [20,21,22,23,24], *Coptis chinensis* [25,26,27] and *Phellodendron chinense* [28]. Therefore HPLC and LC-MS was used to confirm the presence of these alkaloids. Figure 1 illustrates the HPLC profile of the methanol extract of *Coptis chinensis* and lists the alkaloids detected in the different crude extracts of the three most active species. Berberine and palmatine were found in all nine extracts; coptisine only in the crude extracts of *Coptis chinensis*. Total alkaloids were highest in the methanol extract of *Coptis chinensis* and lowest in the aqueous extract of *Berberis bealei*. Berberine is the main alkaloid of all extracts of *Coptis chinensis* and *Phellodendron chinense*. The main alkaloid of *Berberis bealei* is palmatine.

### 2.3. Inhibition of Acetylcholinesterase by Pure Substances

Plants produce a high diversity of secondary metabolites representing a complex mixture of compounds belonging to several chemical classes. The mode of action of most plants cannot be attributed to one single chemical compound, but to the pleiotropic effects of the secondary metabolites contained in the plant [29]. To understand the potential mode of action of the aforementioned TCM plants, the isolated alkaloids berberine, coptisine and palmatine were tested for their individual inhibition of AChE (Table 1). It is notable that none of the three alkaloids inhibits AChE as strong as the crude methanol extract of *Coptis chinensis*.

### 2.4. Inhibition of ACh is Based on Synergism

The finding that none of the tested alkaloids showed an equally strong AChE inhibitory effect as the crude methanol extract of *Coptis chinensis* led to the assumption that the mode of action of this crude extract could be based on synergism of individual alkaloids. Therefore, synergism studies were carried out: the AChE assay was conducted with a dilution series of one of the isolated alkaloids (1st alkaloid) in combination with a steady IC_30_ concentration of the other alkaloids (2nd alkaloid). Data is shown in Table 3. Comparing the FIC values obtained at constant concentrations of the 2nd alkaloid with varying concentrations of the 1st alkaloid showed decreasing FIC values by increasing the concentration of the 1st alkaloid. In some of the tested combinations an FIC value of ≤0.5 was observed, which signifies a synergistic effect.

**Figure 2:**
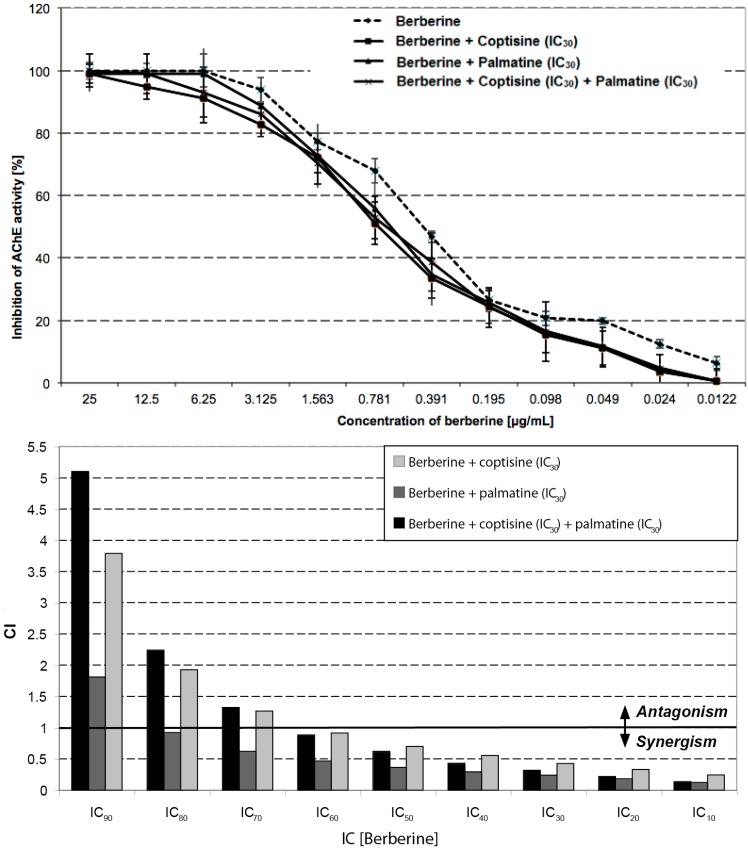
AChE inhibitory effect and synergistic effects of berberine in combination with coptisine and palmatine. The combination of berberine with other alkaloids inhibits ACh stronger than berberine alone (**top**); The combination index (CI) increases with higher concentrations of berberine (**bottom**). Data is shown as mean ± SD from three individual experiments, each carried out in triplicates.

The most apparent synergistic effect was found in the experiments in which coptisine and palmatine were combined with berberine (Figure 2, Table 3). Here, synergy was detected up to IC_60_ concentration of berberine combined with the IC_30_ concentration of coptisine or palmatine. At higher concentrations of berberine, FIC values between 0.5 and 1.0 indicate an additive effect.

In the experiments where coptisine and palmatine were used as 1st alkaloids synergy was only observed at very low concentrations of the 1st alkaloid. Again, FIC values decreased with increasing concentrations of the 1st alkaloid. At high concentrations of the 1st alkaloid even antagonistic effects of the alkaloids analysed were noted.

## 3. Discussion

Nature offers a high diversity of chemical compounds that might be beneficial as potential treatments for human diseases. Therefore, this study aimed at elucidating the potential of 80 TCM plants as inhibitors of acetylcholinesterase, a known Alzheimer target. For that purpose, inhibition of acetylcholinesterase was observed in a high-throughput enzymatic assay. Furthermore, cytotoxicity in COS7 cells was assessed and potential synergistic effects of the chemical compounds contained in the TCM plant extracts were evaluated. In contrast to other research approaches where natural products are used as lead compounds and modified synthetically [30,31] we concentrated on the question if a crude extract comprised of various constituents has benefits over the isolated single components of it.

A range of secondary plant metabolites has shown anti-cholinesterase activity including alkaloids, flavonoids and lignans with alkaloids being the largest group of ACh inhibitors [32,33]. The strong inhibitory activity of alkaloids is associated with their similarity to ACh. Many alkaloids have a positively-charged nitrogen which can bind in the gorge of active site of actetylcholinesterase [34].

Several plant drugs are used to treat deficits in memory and symptoms of AD, including *Coptis chinensis* [25,35], *Magnolia officinalis* [36,37,38], *Cinnamomum cassia* [39] and most commonly, *Ginkgo biloba*. Ginkgo is the most popular plant for the treatment of memory-affiliated problems although no direct anti-acetylcholinesterase activity has been observed so far. In this study none of the crude plant extracts of *Ginkgo biloba* showed any substantial AChE inhibitory activity. The same accounts for *Magnolia officinalis* with only meagre AChE inhibitory activity.

**Figure 3:**
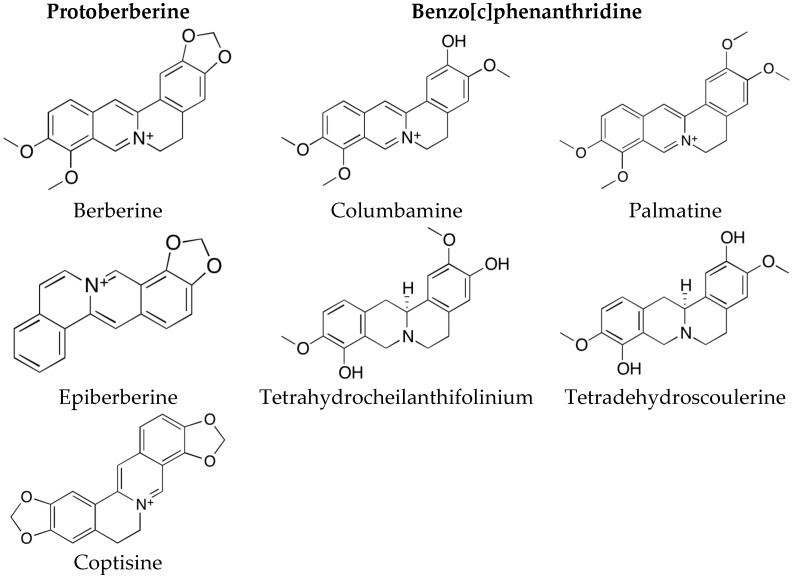
Alkaloids contained in *Coptis chinensis*.

The AChE inhibitory activity of the root of *Coptis chinensis* (Coptidis rhizoma) and its isolated alkaloids has been discussed earlier [25] but the mode of action has not been described so far. Here, a distinctive anti-AChE effect was observed in all three extracts. These effects might be credited to berberine, the main alkaloid of *Coptis chinensis*, as well as the other protoberberine alkaloid coptisine and the benzo[c]phenanthridine alkaloid palmatine (Figure 3). Both the methanol and the aqueous extracts show a stronger inhibition of AChE than galantamine. Remarkably, the methanol extract exhibits an AChE inhibition that is 100-fold stronger than the one observed for galantamine. Usually plants contain a complex profile of secondary metabolites therefore the effect of a plant extract usually cannot be accredited to one single compound. Also synergistic effects have to be taken into account [17,18]. When comparing the IC_50_ values of the crude plant extracts to the IC_50_ values of the pure alkaloids berberine, coptisine and palmatine it strikes out that the methanol extract is much more active than any of the pure alkaloids tested. This suggests that not berberine alone causes the AChE inhibitory activity but rather the combination of alkaloids and other chemical compounds that apparently act synergistically. This study provides evidence that the AChE inhibitory effect of the alkaloids berberine, coptisine and palmatine is clearly synergistic (Table 3, Figure 1). The strongest synergism was observed for the combination of berberine and coptisine. Also the combination of berberine with coptisine and palmatine together produced strong synergistic effects.

LC-MS analysis indicated that in *Coptis chinensis* berberine is the main alkaloid followed by coptisine and palmatine. Therefore, it can be assumed that the strong AChE inhibition of this drug is based on synergistic action of these alkaloids. Furthermore, the methanol extract of *Coptis chinensis* has the highest concentration of alkaloids of all extracts tested, which might explain its extremely strong inhibition of AChE.

*Berberis bealei* comprises of a variety of bioactive secondary metabolites such as protoberberine alkaloids like berberine, columbamine, jatrorrhizine and palmatine [20,21,22,23,24]. All three crude extracts showed an apparent inhibition of AChE. The dichloromethane extract contains the largest amount of alkaloids of the extracts of *Berberis bealei* and also shows the strongest AChE inhibitory activity. The very low IC_50_ of the dichloromethane extract points to the fact that the combination of palmatine and berberine enhances the AChE inhibition. Here, this synergistic effect of this combination is clearly proven but the presence of other compounds like flavonoids or saponins has to be taken into account as well.

The main constituents of *Phellodendron chinense* are isoquinoline alkaloids such as berberine, palmatine, jatrorrhizine and sesquiterpene lactones [28]. So far it can be stated that the active component is largely the alkaloid berberine [40]. All three extracts inhibited AChE substantially. Again, AChE inhibitory activity of these three extracts can be accredited to synergy. In all extracts berberine is the main alkaloid and the synergism experiments hint to the fact that the combination with palmatine increases the AChE inhibitory activity. Compared to *Coptis chinensis*, the AChE inhibitory activity of *Phellodendron chinense* is significantly lower which might be credited to the absence of coptisine in this extract.

Of the TCM plants analysed in this study, *Coptis chinensis, Berberis bealei* and *Phellodendron chinense* seem to be the most promising candidates for an apparent inhibition of AChE activity as all three crude plant extracts show a distinctive inhibitory effect. Most striking of all results is the finding that the methanol extract of *Coptis chinensis* exhibits a 100-fold stronger AChE inhibitory activity than the already known and widely sold AChE inhibitor galantamine, which might be due to the synergistic interaction of the individual alkaloids in this extract.

So far, no data is available about the physiological absorption rate of alkaloids contained in these extracts and it remains unclear if they can pass the blood-brain border. In vivo tests should be carried out to confirm these promising in vitro results in a mouse or rat model of Alzheimer’s Disease.

These findings suggest that TCM plants represent an important source of natural compounds that affect the activity of AChE. Apart from isolating pure compounds as lead structures for novel drugs it might also be possible to administer TCM plant extracts as nutriceuticals. Furthermore, these extracts could be used as cheap alternative to other drugs in third world countries for the treatment of Alzheimer’s Disease.

## 4. Materials and Methods

### 4.1. TCM Plants

All TCM plants were kindly provided by Prof. Thomas Efferth, Johannes Gutenberg University Mainz, Germany and were obtained commercially in China. Identity of the TCM plants was authenticated via DNA barcoding. All samples have accession numbers and voucher specimens were deposited at the IPMB, Department of Biology, Heidelberg University, Germany.

### 4.2. Chemicals

DMEM (Dulbecco’s Modified Eagle’s Medium) media, supplements, fetal bovine serum (FBS), trypsin-EDTA and dimethyl sulphoxide (DMSO) were purchased from Gibco Invitrogen (Karlsruhe, Germany). Berberine, coptisine, galantamine, physostigmine and palmatine were obtained from Fluka/Sigma-Aldrich (Steinheim, Germany).

### 4.3. DNA Barcoding

TCM plants were chosen according to their traditional use. TCM plant samples were obtained commercially. In order to authenticate the plant species, DNA barcoding was carried out to identify the respective species. The plant DNA was isolated from tissue using phenol chloroform extraction protocol [41]. A 700 bp fragment of the chloroplast marker gene rbcL was amplified using PCR.

The PCR products were sequenced and the identity of the plant species was confirmed on either the genus or the species level by comparing the respective sequence with database (NCBI) entries of authentic species.

Clustal W was used to align the sequences [42]; the genetic distances were calculated using MEGA 4.0 following the Kimura 2-Parameter (K2P) model [43]. BLAST database search was performed as described previously [44]; neighbour-joining was used for the phylogenetic tree construction [45].

### 4.4. Preparation of TCM Extracts

50 g finely milled TCM drugs were boiled in reflux in 500 mL of the solvent of choice (methanol, dichloromethane or water) for 6 h. The extract was then filtered through a grade 603 filter. After this, the filtrate was evaporated in a Rotavapor R-200 (Büchi, Flawil, Switzerland). The residual material was resolved in DMSO to a concentration of 50 mg/mL and stored at −20 °C until use.

### 4.5. Cytotoxicity / MTT Assay

COS7 (African green monkey epithelial kidney cells) cells were maintained in DMEM complete media (l-glutamine supplemented with 10% heat-inactivated fetal bovine serum, 5% penicillin/streptomycin). Cells were grown at 37 °C in a humidified atmosphere of 5% CO_2_. All experiments were performed with cells in the logarithmic growth phase. Cytotoxicity of TCM extracts in COS7 cells was determined using different concentrations of extracts. MTT [3-(4,5-dimethylthiazol-2-yl)-2,5-diphenyltetrazolium bromide] was used in a colorimetric assay to determine cell viability and assess cytotoxicity [46]. All experiments were carried out in triplicates and repeated three times. The viability of the cells was determined and data are presented as the percentage of viable cells compared to the control (cells in serum-free medium) in relation to the concentration of the extract.

### 4.6. AChE Assay

An adapted version of the Ellmann assay [47] in 96-well plates was used to measure AChE activity. A mastermix consisting of 25 µL acetylthiocholine iodide (ACTI, 15 mM in phosphate buffer pH 7), 125 µL dithionitrobenzoic acid (DTNB, 3 mM in phosphate buffer pH 8) and 50 µL phosphate buffer (50 mM, pH 8) was prepared and added to 5 µL of TCM crude plant extract (stock 50 mg/mL) or 5 µL essential oil per well. It turned out to be crucial to prepare all reaction solutions freshly for every set of experiments. Two known AChE inhibitors, physostigmine and galantamine (50 mg/mL in phosphate buffer pH 8), served as positive controls. As all crude extracts (water, methanol, dichloromethane) were evaporated and then resolved in DMSO beforehand, DMSO was used as negative control in all experiments.

After shaking for 20 s, measurements at time *t* = 0, 3, 6, and 9 min were recorded at 450 nm using the EL808 plate reader (BioTek, Winooski, VT 05404, United States) to avoid interference of results due to spontaneous activity. After 9 min reading, 25 µL of acetylcholinesterase (AChE from electric eel, 3 U/mL in phosphate buffer pH 8) were added to each well and the plate was left to incubate at room temperature for 3 min. After shaking for 20 s a final reading was recorded at 450 nm. The inhibitory activity was calculated in comparison to the negative control. Potential effects were expressed as the percentage of inhibition. The experiments were carried out three times and all samples were measured three times. For the most active *Coptis chinensis* samples the experiment was carried out nine times to corroborate that the findings were correct.

### 4.7. Phytochemical Analysis

The most active extracts were analysed phytochemically by high performance liquid chromatography (HPLC) and mass spectrometry (MS).

HPLC: A Merck Hitachi HPLC system (Merck, Darmstadt, Germany) composed of a binary L-6200A intelligent pump and an ERC-3215α degasser was used. The extracts, with a final concentration of 500 µg/mL in methanol, were injected in the HPLC system via a Rheodyne system with a 20 µL loop. Separation was achieved using a RP-C18e Lichrospher 100 (250 mm length, 4 mm diameter column, 5 µm particle size) (Merck, Darmstadt, Germany). The mobile phase consisted of: A: Water HPLC grade with 0.5% formic acid and ammonium acetate pH = 7; B: acetonitrile. The gradient program at a flow rate of 1 mL/min as following: 0% to 40% B in 15 min, then to 100% B in 10 min, then in 5 min. back to the initial condition. A mechanical split with 10% to the MS machine and 90% as waste was used after the separation column.

MS: A Quattro II system (Waters, Eschborn, Germany) from VG with an ESI interface was used in the positive ion mode under the following conditions: Drying and nebulizing gas: N_2_, capillary temperature: 120 °C; capillary voltage: 3.50 kV; lens voltage: 0.5 kV; cone voltage: 30 V. Scan mode at range *m*/*z* 300–600. Chromatograms were processed using Waters MassLynx software (Version 4.0, Waters, Eschborn, Germany).

### 4.8. Evaluation of Synergistic Effects

After screening the TCM crude extracts and their chemical compounds it became clear that the distinctive inhibitory effect of some of the crude extracts must be based on synergism of individual isoquinoline alkaloids in the extracts. Therefore, synergism studies were carried out with isoquinoline alkaloids berberine, coptisine and palmatine. First the IC_30_ values for all samples were calculated. For “compound A” a serial dilution was prepared and seeded to the 96-well plate. “Compound B” was added to each well at a fixed concentration that corresponded to its IC_30_ value. After this the AChE inhibition assay was carried out as described before. All possible combinations of diluted “compound A” and fixed “compound B” were analysed.

Drug interaction was classified as either synergistic, additive indifferent or antagonistic based on the fractional inhibitory concentration (FIC) index. The fractional effect (FE) of two compounds is calculated from the effect caused by two compounds in combination in relation to the effect of one compound alone: FEa = IC_50_(a + b)/IC_50_(a); FEb = IC_50_(a + b)/IC_50_(b). By plotting these values against each other the isobologram showing the areas of synergy is obtained.

The FIC index is the sum of both FE indexes. According to Berenbaum [48], FIC ≤ 1.0 signifies synergy, FIC = 1.0 an additive effect and FIC ≥ 1.0 antagonism. Schelz [49] regards FIC ≤ 0.5 as synergy, FIC > 0.5 to 1.0 as additive, FIC = 1.0 to 4.0 as indifferent and FIC ≥ 4.0 as antagonism. We were following the second perspective.

### 4.9. Statistical Analysis

All experiments were carried out in triplicates and repeated at three individual days. All data are expressed as mean ± standard deviation (*n* = 3). The IC_50_ values were calculated using a four-parameter logistic curve (SigmaPlot^®^ 11.0) representing 50% reduction of activity. Statistical analysis of the effects of increasing concentrations of samples on the viability of COS7 cells and activity of AChE was performed using Student’s t-test in SigmaPlot^®^ 11.0 (Systat, Erkrath, Germany) to determine significance of the difference between two data sets. The significance level of *p* < 0.05 denotes significance for all cytotoxicity and AChE experiments.

## 5. Conclusions

Various plant-derived compounds are already used for the treatments of Alzheimer’s Disease indicating that nature is a valuable source of new bioactive agents. We tested various plants from Traditional Chinese Medicine for their potential inhibition of AChE activity. Based on our results it can be stated that some TCM plants inhibit AChE via synergistic interaction of their secondary metabolites. The possibility to isolate pure lead compounds from the crude extracts or to administer these as nutraceuticals or as cheap alternative to drugs in third world countries make TCM plants a versatile source of natural inhibitors of AChE.

## Figures and Tables

**Table 1:** AChE inhibitory (AChEi) activity and cytotoxicity in COS7 cells of the most active TCM plant extracts. All data are expressed as mean ± standard deviation; all experiments were carried out in triplicates and repeated independently. (AChE assay: *n* = 3; *n* = 9 for *Coptis chinensis* samples. Cytotoxicity: *n* = 3).

Sample	IC_50_ AChE Inhibition (mg/mL)	IC_50_ COS7 (µg/mL)	Ratio IC_50_ COS7/AChE
*Berberis bealei* MeOH	34.10 ± 4.89	35.37 ± 4.21	1.0
*Berberis bealei* CH_2_Cl_2_	9.99 ± 1.18	13.36 ± 1.76	1.3
*Berberis bealei* H_2_O	87.77 ± 4.11	270.0 ± 13.5	3.1
*Coptis chinensis* MeOH	0.031 ± 0.002	3.72 ± 0.74	120
*Coptis chinensis* CH_2_Cl_2_	8.13 ± 0.90	39.57 ± 4.87	4.9
*Coptis chinensis* H_2_O	2.5 ± 0.61	118.3 ± 7.4	47
*Phellodendron chinense* MeOH	8.03 ± 0.98	85.52 ± 11.90	10
*Phellodendron chinense* CH_2_Cl_2_	6.34 ± 1.37	71.33 ± 6.87	11
*Phellodendron chinense* H_2_O	84.83 ± 1.84	282.9 ± 15.3	3.3
Berberine	1.48 ± 0.07	-	-
Coptisine	1.27 ± 0.06	-	-
Palmatine	5.21 ± 0.48	-	-
Physostigmine	2.24 ± 0.27	-	-
Galantamine	4.33 ± 0.21	-	-

**Table 2:** AChE inhibitory activity and cytoxicity in COS7 cells of TCM plant extracts.

Family/Plant	Chinese Name	DNA	AChE (IC_50_ (µg/mL))			COS7 (IC_50_ (µg/mL))		
			MeOH	CH_2_Cl_2_	H_2_O	MeOH	CH_2_Cl_2_	H_2_O
Acanthaceae	-	-	-	-	-	-	-	-
*Andrographis paniculata*	Chuan Xin Lian	Family	NA	NA	NA	344.7 ± 13.3	104.7 ± 5.2	255.6 ± 11.9
Amaranthaceae	-	-	-	-	-	-	-	-
*Celiosa cristata*	Ji Guan hua	Genus	NA	NA	NA	28.43 ± 2.87	136.00 ± 8.40	263.9 ± 12.3
Apiaceae	-	-	-	-	-	-	-	-
*Bupleurum chinense*	Chai Hu	Genus	NA	NA	NA	358.7 ± 19.8	87.12 ± 3.67	15.60 ± 11.76
*Bupleurum marginatum*	Nan Chai Hu	Genus	NA	NA	NA	576.0 ± 31.2	67.41 ± 5.23	350.7 ± 17.5
*Centella asiatica*	Lei Gong Gen	Species	NA	NA	NA	392.8 ± 78.2	64.97 ± 4.85	325.8 ± 12.1
*Saposhnikovia divaricata*	Fang Feng	Genus	NA	NA	NA	1575 ± 147	46.00 ± 2.64	153.0 ± 7.7
*Selinum monnieri*	She Chuang Zi	Family	NA	NA	NA	120.0 ± 11.7	37.02 ± 2.39	339.7 ± 11.1
Araliaceae	-	-	-	-	-	-	-	-
*Eleutherococcus senticosus*	Ci Wu Jia	Species	NA	NA	NA	190.1 ± 18.4	61.49 ± 5.98	130.5 ± 7.5
*Panax ginseng*	Ren Shen	Species	NA	NA	NA	510.8 ± 29.5	47.76 ± 5.82	151.7 ± 8.9
*Panax notoginseng*	San Qi	Species	NA	NA	NA	229.5 ± 19.5	6.47 ± 0.89	182.3 ± 9.1
Arecaceae	-	-	-	-	-	-	-	-
*Areca catechu*	Bing Lang	n/a	NA	NA	NA	31.02 ± 2.69	117.02 ± 7.33	16.60 ± 2.01
Apogynaceae	-	-	-	-	-	-	-	-
*Cyanchum paniculatum*	Liao Diao Zhu	Genus	NA	NA	NA	227.7 ± 16.9	114.25 ± 6.78	220.7 ± 7.6
Asparagaceae	-	-	-	-	-	-	-	-
*Polygonatum humile*	Huan Jjing	Species	NA	NA	NA	147.4 ±15.4	53.94 ± 4.67	298.4 ± 13.6
Asteraceae	-	-	-	-	-	-	-	-
*Arcticum lappa*	Niu Bang	Species	NA	NA	NA	1813 ± 225	344.25 ± 12.31	355.5 ± 12.6
*Artemisia annua*	Huang Hua Hao	n/a	NA	NA	NA	201.1 ± 8.7	34.57 ± 3.18	288.6 ± 11.4
*Artemisia capillaris*	Yin Chen Hao	Genus	NA	NA	NA	215.4 ± 9.3	29.49 ± 2.56	201.9 ± 9.5
*Centipeda minima*	Ebu Shi Cao	n/a	NA	NA	NA	54.21 ± 4.98	10.44 ± 1.70	55.64 ± 4.62
*Chrysanthemum indicum*	Ye Ju Hua	Genus	NA	NA	NA	287.2 ± 9.8	63.58 ± 5.78	320.9 ± 14.3
*Chrysanthemum morifolium*	Ju Hua	Genus	NA	NA	NA	166.7 ± 8.4	42.88 ± 3.96	760.4 ± 28.3
*Eclipta prostata*	Han Lian Cao	Species	NA	NA	NA	186.1 ± 12.8	112.06 ± 9.65	291.7 ± 13.9
*Senecio scandens*	Qian Li Guang	Genus	NA	NA	NA	126.2 ± 12.2	143.54 ± 9.64	114.2 ± 6.7
*Siegesbeckia orientalis*	Xi Xian Cao	Family	NA	NA	NA	84.4 ± 7.5	17.78 ± 1.94	159.2 ± 7.4
*Taraxum officinale*	Pu Gong Ying	Species	NA	NA	NA	485.3 ± 17	177.16 ± 8.45	156.9 ± 6.3
Berberidaceae	-	-	-	-	-	-	-	-
*Berberis bealei*	Shi Da Gong Lao	Species	34.10 ± 4.89	9.99 ± 1.18	87.77 ± 4.11	35.37 ± 4.21	13.36 ± 1.76	270.0 ± 13.5
*Dysosma versipellis*	Ba Jiao Lian	n/a	NA	NA	NA	54.90 ± 4.69	49.95 ± 5.29	1276 ± 39
*Epimedium koreanum*	Yin Yang Huo	Species	NA	NA	NA	0.37 ± 0.03	3.60 ± 0.56	140.8 ± 6.2
Brassicaceae	-	-	-	-	-	-	-	-
*Isatis indigotica rhizome*	Ban Langen	Family	NA	NA	NA	324.4 ± 17.8	42.38 ± 4.54	557.2 ± 27.2
*Isatis indigotica leaf*	Daq Qing Ye	Family	NA	NA	NA	90.62 ± 11.37	0.64 ± 0.07	93.51 ± 4.73
*Capsella bursa-pastoris*	Ji Cai	Species	NA	NA	NA	29.82 ± 3.87	120.82 ± 7.89	234.2 ± 11.6
Caprifoliaceae	-	-	-	-	-	-	-	-
*Lonicera confusa*	Ren Dong Teng	Genus	NA	NA	NA	118.9 ± 12.8	58.99 ± 5.13	446.8 ± 21.2
Crassulacea	-	-	-	-	-	-	-	-
*Sedum rosea*	Hong Jing Tian	Species	NA	NA	NA	87.42 ± 7.43	74.67 ± 6.54	61.97 ± 4.12
Cupressaceae	-	-	-	-	-	-	-	-
*Platycladus orientalis*	Ce Bai Ye	Species	NA	NA	NA	158.7 ± 12.4	21.80 ± 2.91	97.78 ± 56.53
Dryopteridaceae	-	-	-	-	-	-	-	-
*Cyrtomium fortunei*	Guang Zhong	Species	NA	NA	NA	348.7 ± 26.5	132.13 ± 5.03	30.42 ± 2.45
Ephedraceae	-	-	-	-	-	-	-	-
*Ephedra sinica*	Ma Huang	Species	NA	NA	NA	36.76 ± 3.93	41.82 ± 4.85	69.15 ± 5.98
Equisetaceae	-	-	-	-	-	-	-	-
*Equisetum hiemale*	Mu Zei	Species	NA	NA	NA	243.5 ± 17.1	35.76 ± 3.50	265.9 ± 12.3
Euphorbiaceae	-	-	-	-	-	-	-	-
*Croton tiglium*	Ba Dou	n/a	NA	NA	NA	222.1 ± 18.4	225.98 ± 10.69	166.2 ± 5.7
Fabaceae	-	-	-	-	-	-	-	-
*Abrus cantonensis*	Ji Gu Cao	Genus	NA	NA	NA	733.1 ± 42.6	129.45 ± 7.65	575.2 ± 24.4
*Acacia catechu*	Er Cha	n/a	NA	NA	NA	34.86 ± 2.55	31.56 ± 3.78	35.71 ± 3.86
*Cassia tora*	Jue Ming Zi	Genus	NA	NA	NA	75.95 ± 6.50	189.15 ± 8.43	481.3 ± 19.4
*Desmodium styracifolium*	Guang Jin Qian Cao	Genus	NA	NA	NA	104.1 ± 9.35	139.86 ± 5.50	333.5 ± 13.9
*Glycyrrhiza inflata*	Gan Cao	Species	NA	333 ± 9	NA	126.8 ± 11.2	6.97 ± 1.43	583.9 ± 21.3
*Spatholobus suberectus*	Ji Xue Teng	Genus	NA	NA	NA	54.87 ± 4.89	154.66 ± 6.72	16.63 ± 1.32
*Sutherlandia frutescens*	n/a	n/a	NA	NA	NA	352.0 ± 29.5	259.37 ± 9.39	857.3 ± 26.8
Geraniaceae	-	-	-	-	-	-	-	-
*Geranium wilfordii*	Loa Guan Cao	Genus	NA	NA	NA	169.8 ± 16.4	17.02 ± 1.80	225.8 ± 9.6
Ginkgoaceae	-	-	-	-	-	-	-	-
*Ginkgo biloba*	Yin Xing	Species	NA	1003 ± 15	NA	260.1 ± 24.21	15.40 ± 1.34	450.8 ± 21.3
Hypericaceae	-	-	-	-	-	-	-	-
*Hypericum japonicum*	Tian Ji Huang	Genus	NA	NA	NA	100.9 ± 9.0	10.83 ± 0.53	151.8 ± 13.9
Iridaceae	-	-	-	-	-	-	-	-
*Belamcanda chinensis*	She Gan	Species	NA	NA	NA	319.5 ± 28.4	89.25 ± 7.32	222.1 ± 12.7
Lamiaceae	-	-	-	-	-	-	-	-
*Mentha haplocalyx*	Bo He	Genus	NA	NA	NA	147.8 ± 12.3	34.16 ± 3.35	285.7 ± 14.6
*Prunella vulgaris*	Xia Ku Cao	Species	NA	NA	NA	494.5 ± 45.7	90.48 ± 6.47	21.57 ± 2.90
*Scutellaria baicalensis*	Huang Qin	Species	NA	NA	NA	28.88 ± 2.65	287.98 ± 8.93	46.41 ± 3.96
Lauraceae	-	-	-	-	-	-	-	-
*Cinnamomum cassia*	Gui Zhi	Genus	1027 ± 16	953 ± 12	NA	108.4 ± 9.8	23.20 ± 2.76	453.9 ± 21.5
Loranthaceae	-	-	-	-	-	-	-	-
*Taxillus chinensis*	Sang Ji Sheng	Family	NA	NA	NA	378.2 ± 32.4	68.65 ± 7.42	181.7 ± 9.2
Lythraceae	-	-	-	-	-	-	-	-
*Punica granatum*	Shi Liu	Species	NA	NA	NA	218.6 ± 16.3	126.64 ± 8.64	8.608 ± 1.432
Magnoliaceae	-	-	-	-	-	-	-	-
*Magnolia officinalis*	Hou Pu	Species	320 ± 9	183 ± 6	NA	13.12 ± 0.97	5.45 ± 1.37	73.01 ± 5.42
Melanthiaceae	-	-	-	-	-	-	-	-
*Paris polyphylla*	Qi Ye Yi Zhi Hua	Species	NA	NA	NA	5.52 ± 0.39	24.07 ± 2.82	38.49 ± 2.47
Myrisinaceae	-	-	-	-	-	-	-	-
*Lysimachia christinae*	Jin Qian Cao	Genus	NA	NA	NA	436.3 ± 36.6	137.39 ± 6.39	152.2 ± 8.4
Myrtaceae	-	-	-	-	-	-	-	-
*Eucalyptus robusta*	An Shu	n/a	NA	NA	NA	15.21 ± 0.62	n/a	94.19 ± 5.99
Oleaceae	-	-	-	-	-	-	-	-
*Fraxinus chinensis*	Qin Pi	n/a	NA	NA	NA	38.72 ± 6.59	39.23 ± 4.11	193.2 ± 11.6
Ophioglossacea	-	-	-	-	-	-	-	-
*Ophioglossum vulgatum*	Yi Zhi Jian	Species	NA	NA	NA	68.70 ± 11.42	62.87 ± 6.58	344.0 ± 13.4
Orchideaceae	-	-	-	-	-	-	-	-
*Dendrobium loddigesii*	Shi Hu	Species	NA	NA	NA	61.65 ± 15.36	25.74 ± 2.94	104.3 ± 6.3
Paeoniaceae	-	-	-	-	-	-	-	-
*Paeonia lactiflora*	Chi Shao	Genus	NA	NA	NA	309.8 ± 24.7	34.06 ± 4.60	148.2 ± 6.1
Pedaliaceae	-	-	-	-	-	-	-	-
*Harpagophytum procumbens*	n/a	n/a	NA	NA	NA	217.2 ± 18.53	15.89 ± 2.25	242.9 ± 12.6
Poaceae	-	-	-	-	-	-	-	-
*Cymbopogon distans*	Yun Xian Cao	Genus	NA	NA	NA	17.88 ± 1.16	114.57 ± 8.63	257.8 ± 13.2
Polygonaceae	-	-	-	-	-	-	-	-
*Fallopia multiflora*	He Shou Wu	Genus	NA	523 ± 11	NA	48.87 ± 4.62	107.74 ± 7.94	61.32 ± 5.61
*Polygonum cuspidatum*	Hu Zhang	Species	NA	NA	NA	19.59 ± 2.23	2.85 ± 0.87	39.81 ± 3.84
*Rheum officinale*	Da Huang	Species	NA	NA	NA	3.53 ± 0.80	n/a	51.59 ± 5.48
Ranunculaceae	-	-	-	-	-	-	-	-
*Coptis chinensis*	Huang Lian	Species	0.031 ± 0.002	8.13 ± 0.90	2.5 ± 0.61	3.72 ± 0.74	39.57 ± 4.87	118.3 ± 7.4
Rosaceae	-	-	-	-	-	-	-	-
*Rosa chinensis*	Yu Ji Hua	n/a	NA	NA	NA	36.76 ± 3.45	141.55 ± 9.52	24.32 ± 2.86
*Rosa laevigata*	Jin Ying Zi	n/a	NA	NA	NA	13.30 ± 2.21	151.80 ± 9.79	93.61 ± 7.30
*Sanguisorba officinalis*	Di Yu	Species	NA	NA	NA	1100 ± 97	26.77 ± 3.58	20.55 ± 2.59
Rubiaceae	-	-	-	-	-	-	-	-
*Hedyotis diffusa*	Bai Hua She She Cao	Genus	NA	NA	NA	418.1 ± 34.8	45.35 ± 4.12	158.7 ± 9.2
Rutaceae	-	-	-	-	-	-	-	-
*Evodia lepta*	San Cha Ku	Family	NA	NA	NA	5.13 ± 0.75	42.06 ± 4.65	419.3 ± 19.4
*Evodia rutaecarpa*	Wu Zhu Yu	n/a	NA	NA	NA	427.7 ± 37.2	8.78 ± 1.78	1176 ± 34
*Phellodendron chinense*	Huang Bai	Genus	8.03 ± 0.98	6.34 ± 1.37	84.83 ± 1.84	85.52 ± 11.90	71.33 ± 6.87	282.9 ± 15.3
Saururaceae	-	-	-	-	-	-	-	-
*Houttuynia cordata*	Yu Xing Cao	Species	NA	NA	NA	63.95 ± 5.98	48.26 ± 5.19	633.3 ± 17.8
Schisandraceae	-	-	-	-	-	-	-	-
*Kadsura longipedunculata*	Zi Ging Pi	Species	NA	NA	NA	43.92 ± 2.59	1.88 ± 0.94	6.812 ± 1.678
Selaginellaceae	-	-	-	-	-	-	-	-
*Selaginella tamariscina*	Juan Bai	Species	NA	NA	NA	150.9 ± 13.34	98.83 ± 8.62	103.9 ± 6.
Valerianaceae	-	-	-	-	-	-	-	-
*Patrinia scabiosaefolia*	Bai Jiang	Genus	NA	NA	NA	15.96 ± 2.69	38.77 ± 2.87	38.49 ± 3.73
Verbenaceae	-	-	-	-	-	-	-	-
*Verbena officinalis*	Ma Bian Cao	Species	NA	NA	NA	37.76 ± 4.42	145.91 ± 9.06	93.94 ± 5.97
Violaceae	-	-	-	-	-	-	-	-
*Viola yezoensis*	Zi Hua Di Ding Cao	Genus	NA	NA	NA	19.19 ± 1.45	60.87 ± 5.49	135.6 ± 6.4
Zingiberaceae	-	-	-	-	-	-	-	-
*Alpinia galanga*	Hong Dou Kou	n/a	NA	NA	NA	53.48 ± 4.92	5.76 ± 1.53	952.2 ± 31.2
*Alpinia oxaphylla*	Yi Zhi Ren	Species	NA	NA	NA	110.2 ± 9.5	30.40 ± 2.81	105.8 ± 7.5

All data are expressed as mean ± standard deviation; all experiments were carried out in triplicates and repeated independently. (AChE assay: *n* = 3; *n* = 9 for *Coptis chinensis* samples. Cytotoxicity: *n* = 3). Samples were considered to be inactive (NA) in the AChE assay if they showed less than 80% inhibition of AChE activity at a concentration of 1250 µg/mL. For some plants not all extracts could be prepared, these samples are marked n/a (not analysed).

**Table 3:** AChE inhibitory activity of combinations of berberine, coptisine and palmatine. **T**he 1st alkaloid (italics) was diluted in 1:1 steps; the 2nd alkaloid was used in a steady IC_30_ concentration. All IC values are stated in (µg/mL). A combination index (CI) < 1.0 (bold) indicates synergism. All data is shown as mean ± SD from three independent experiments, each carried out as a triplicate.

Sample	IC_10_	IC_20_	IC_30_	IC_40_	IC_50_	IC_60_	IC_70_	IC_80_	IC_90_
***Berberine***	0.27	0.51	0.76	1.08	1.48	2.02	2.85	4.32	8.09
*Berberine* + coptisine IC_30_	0.051	0.11	0.18	0.28	0.4	0.59	0.9	1.5	3.22
CI	**0.24**	**0.33**	**0.43**	**0.55**	**0.7**	**0.91**	1.26	1.92	3.79
*Berberine* + palmatine IC_30_	0.031	0.08	0.15	0.24	0.37	0.59	0.96	1.75	4.33
CI	**0.12**	**0.18**	**0.24**	**0.29**	**0.36**	**0.47**	**0.62**	**0.92**	1.81
*Berberine* + coptisine IC_30_ + palmatine IC_30_	0.027	0.066	0.12	0.19	0.31	0.48	0.78	1.42	3.47
CI	**0.14**	**0.22**	**0.32**	**0.43**	**0.62**	**0.88**	1.32	2.24	5.1
***Coptisine***	0.6	0.79	0.95	1.11	1.27	1.46	1.69	2.04	2.68
*Coptisine* + berberine IC_30_	0.12	0.26	0.44	0.67	1	1.49	2.3	3.91	8.67
CI	**0.36**	**0.67**	1.04	1.49	2.11	2.98	4.39	7.06	14.6
*Coptisine* + palmatine IC_30_	0.13	0.3	0.52	0.82	1.23	1.86	2.9	5.01	11.4
CI	**0.25**	**0.47**	**0.7**	**0.98**	1.33	1.82	2.57	3.93	7.6
*Coptisine* + berberine IC_30_ + palmatine IC_30_	0.13	0.29	0.47	0.73	1.08	1.6	2.44	4.11	8.97
CI	**0.43**	**0.83**	1.25	1.83	2.59	3.67	5.37	8.63	17.8
***Palmatine*** (µg/mL)	1.73	2.6	3.4	4.25	5.21	6.39	7.97	10.4	15.7
*Palmatine* + berberine IC_30_	0.25	0.67	1.28	2.18	3.54	5.77	9.81	18.7	49.6
CI	**0.47**	1.14	2.06	3.38	5.34	8.49	14.1	26.4	68.5
*Palmatine* + coptisine IC_30_	0.37	0.96	1.82	3.08	5	8.11	13.7	26.1	68.8
CI	**0.44**	**0.76**	1.1	1.49	1.97	2.6	3.54	5.13	8.99
*Palmatine* + berberine IC_30_ + coptisine IC_30_	0.43	1.06	1.95	3.2	5.05	7.97	13.1	24	59.8
CI	1.27	2.92	5.19	8.33	12.9	20.12	32.7	59.2	145
